# Baicalin ameliorates neuropathology in repeated cerebral ischemia-reperfusion injury model mice by remodeling the gut microbiota

**DOI:** 10.18632/aging.102846

**Published:** 2020-02-21

**Authors:** Jianfeng Liu, Tianhua Zhang, Yingying Wang, Chengqing Si, Xudong Wang, Rui-Tao Wang, Zhonghua Lv

**Affiliations:** 1 Department of Neurology, The First Affiliated Hospital of Harbin Medical University, Harbin 150001, China; 2 Department of Vascular Surgery, The Second Affiliated Hospital of Harbin Medical University, Harbin 150086, China; 3 Department of Computer Science, Hong Kong Baptist University, Hong Kong, China; 4 Department of Internal Medicine, Harbin Medical University Cancer Hospital, Harbin Medical University, Harbin 150081, China; 5 Department of Neurosurgery, Harbin Medical University Cancer Hospital, Harbin Medical University, Harbin 150081, China

**Keywords:** baicalin, cerebral ischemia-reperfusion, cognitive behavior, gut microbiota

## Abstract

We investigated the neuroprotective effects of baicalin and the role of gut microbiota in a mouse model of cerebral ischemia-reperfusion injury. Repeated cerebral ischemia-reperfusion significantly increased plasma levels of trimethylamine (TMA), trimethylamine-*N*-oxide (TMAO), and clusterin (a neuroinflammation biomarker). These changes correlated with cognitive decline; short-term memory deficits; abnormal long term potentiation (LTP); decreased functional connectivity (FC) between various brain regions; reduced plasticity and dendritic spine density in the hippocampus; increased levels of the pro-inflammatory cytokines IL-1β, IL-6, and TNFα; and altered the gut microbial composition. Treatment with 50-100 mg/Kg baicalin for 7 days after cerebral ischemia-reperfusion significantly restored normal plasma levels of TMA, TMAO, and clusterin. Baicalin treatment also suppressed neuroinflammation, remodeled the gut microbial composition back to normal, and improved cognition, memory, LTP, cerebral FC, and hippocampal neuronal plasticity. The neuroprotective effects of baicalin were diminished when mice undergoing repeated cerebral ischemia-reperfusion were pretreated with broad-spectrum antibiotics to deplete gut microbial populations. This suggests the neuroprotective effects of baicalin in cerebral ischemia-reperfusion injury are mediated by the gut microbiota. It thus appears that baicalin ameliorates neuropathology in a repeated cerebral ischemia-reperfusion model mice by remodeling the gut microbiota.

## INTRODUCTION

Ischemic stroke accounts for approximately 87% of all reported stroke cases and is frequently associated with cognitive dysfunction [1, 2]. Currently, the standard treatment for acute ischemic stroke is intravenous thrombolytic therapy with recombinant tissue plasminogen activator (rt-PA) [3]. However, the drawbacks of rt-PA therapy include adverse reactions to intravenous injections and narrow time window for thrombolysis [4]. There is no alternate medication for stroke patients, especially those who have previously suffered cerebral ischemia. Hence, there is an urgent need for a novel, effective, and safe therapy for cerebral ischemia-reperfusion injury.

Several studies have highlighted extensive interaction between the host and intestinal flora. The status of gut function and the composition of the gut microbiota can influence brain function and diseases [5, 6]. Cerebral infarction in cynomolgus monkeys causes long-term dysbiosis of the gut microbiota and systemic neuroinflammation [7]. In the mouse model of cerebral hypoperfusion, consumption of multistrain probiotics can alleviate hippocampal injury and deficits in spatial and learning memory, thereby providing more evidence for the key role of gut microbiota in brain function [8]. Cerebral ischemia-reperfusion injury induces dysbiosis of the gut microbiota, which alters the immune homeostasis in the small intestine and modulates neuroinflammation and brain functions [9–11]. Moreover, depletion of gut microbiota with broad-spectrum antibiotic pre-treatment worsens the outcomes of stroke in the mouse model of cerebral ischemia-reperfusion injury [12]. These results suggest that maintaining the homeostasis of the intestinal microflora is critical in ameliorating brain function after brain ischemia-reperfusion injury.

The Chinese traditional medicine, Huang-qin is prepared from the roots of *Scutellaria baicalensis* and has been used since 2000 years to treat influenza, pneumonia, dysentery, and cancer in China [13]. Baicalin is one of the active flavonoids in Huang-qin with anti-tumor, anti-viral, anti-microbial, anti-inflammatory, antioxidative, and neuroprotective properties. *In vitro* cell culture experiments and animal model studies demonstrate that baicalin protects against cerebral ischemia-reperfusion injury and modulates brain function by inhibiting the phosphorylation of CaMKII [14] and maintaining mitochondrial homeostasis [15]. Baicalin improves glycolipid metabolism and the production of short-chain fatty acids by remodeling the gut microbiota [16]. The mechanistic details underlying the improvements in cognition and memory deficits through baicalin-related gut microbial remodeling are not known in the context of repeated cerebral ischemia-reperfusion. Furthermore, the relationship between cognitive changes, composition of the gut microbiota, and ischemia/reperfusion is not well understood. Therefore, we investigated the neuroprotective mechanism of orally administered baicalin in the mouse model of repeated cerebral ischemia-reperfusion injury, including the effects of baicalin on the gut microbiota.

## RESULTS

### Repeated cerebral ischemia-reperfusion causes cognitive impairment and long-term potentiation (LTP) decline in mice

**Figure 1:**
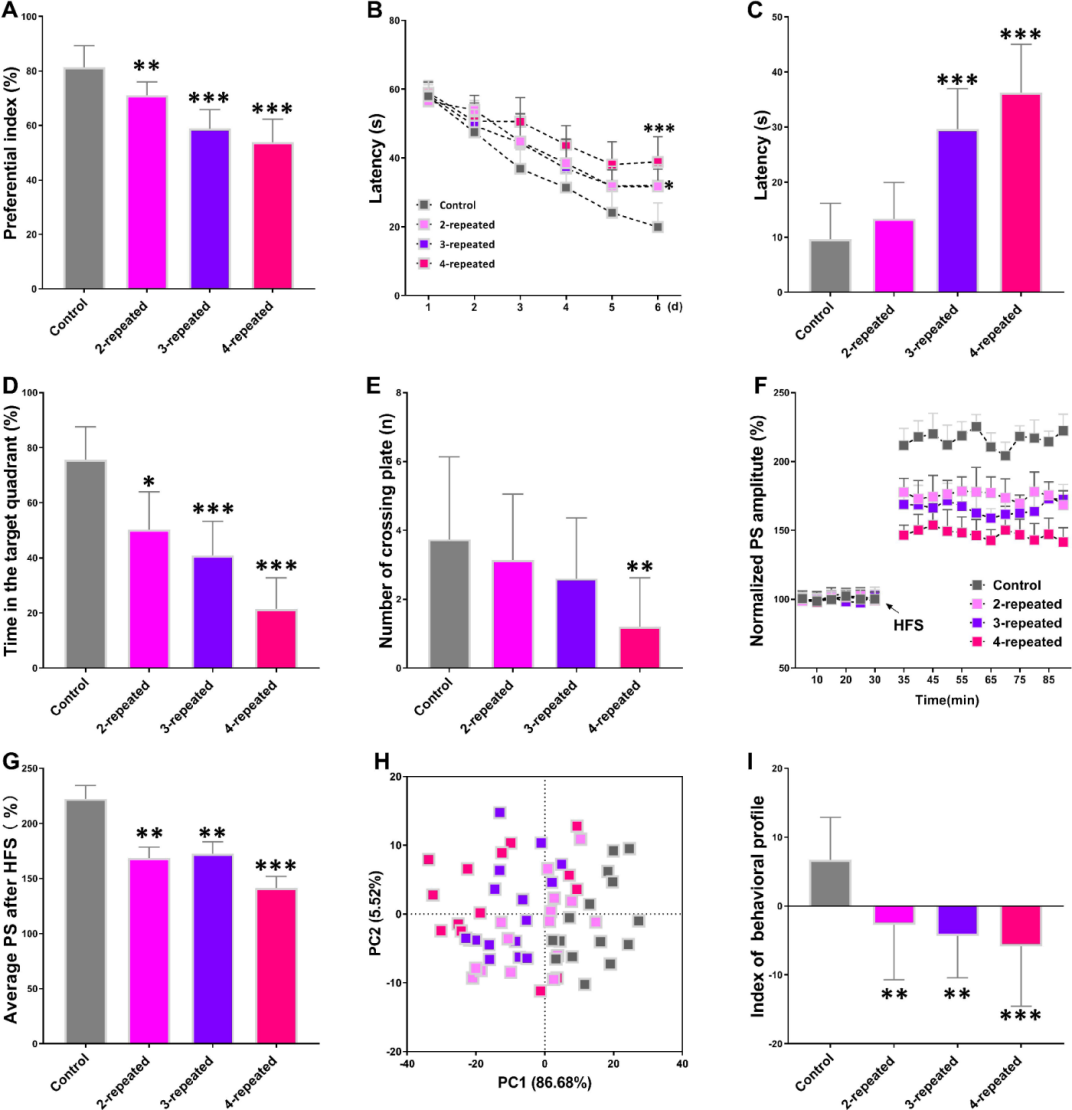
**Repeated cerebral ischemia-reperfusion causes cognitive, memory, and long-term potentiation (LTP) decline in mice.** (**A**–**C**) The novel object response test results show (**A**) preferential index after 1h training and latency in the (**B**) learning and (**C**) testing phases for control and repeated cerebral ischemia-reperfusion injury model group mice. (**D**, **E**) Morris water maze test results show (**D**) time spent in the target quadrant and (**E**) the number of crossing plate in the testing phase for control and repeated cerebral ischemia-reperfusion model group mice. (**F**) Population spike amplitudes and LTP induction is shown for control and repeated cerebral ischemia-reperfusion model mice. (**G**) The average population spike amplitudes are shown for the control and repeated cerebral ischemia-reperfusion model group mice. (**H**) Principal component analysis (PCA) of the data from behavioral and electrophysiological experiments in the control and repeated cerebral ischemia-reperfusion is shown. Each dot represents one mouse. (**I**) The mean PCA scores with or without choline are shown for control and repeated cerebral ischemia-reperfusion model group mice. Note: * denotes *P*<0.05, ** denotes *P*<0.01, and *** denotes *P*<0.001 in comparison with control group mice. The statistical analysis was performed by one-way ANOVA followed by Dunnett’s post hoc test or a two-way repeated-measures ANOVA with post-hoc Tukey multiple comparisons test. All the values are expressed as means ± S.D. Each group had 15 mice (n=15).

The object recognition test results showed that the preferential index after training for 1h was significantly reduced in the mice subjected to repeated cerebral ischemia-reperfusion (model group) compared with the control mice (Figure 1A). The Morris water maze test results showed longer escape latency for model group mice than the control mice during the training and testing periods (Figure 1B and 1C). Moreover, the model group mice showed reduced swimming time within the target quadrant compared to the control mice (Figure 1D). However, there were no significant differences between the control and model groups of mice in the number of plate crossings (Figure 1E) and swimming speed (data not shown). Long term potentiation (LTP) is the most probable neuromechanism for memory formation and storage, and cognition. The average population spike (PS) amplitude after high-frequency stimulation (HFS) was significantly reduced in the model group mice compared to the control mice (Figure 1F and 1G). This suggests deficits in long term potentiation (LTP), which is responsible for cognition and memory.

We then performed the principal component analysis (PCA) using the results of novel object recognition, Morris water maze, and LTP tests to determine the association between repeated cerebral ischemia-reperfusion and cognitive decline. The plot of principal component 1 (PC1, 86.68%) vs. principal component 2 (PC2, 5.52%) grouped the mice into four distinct segments, with the control group in the bottom-right, the 4-repeat group in the top-left, and the 2-repeat and 3-repeat groups in between the control and the 4-repeat groups (Figure 1H). Furthermore, the average PCA score showed that the behavioral and electrophysiological characteristics of the model group mice were significantly different from the control mice (Figure 1I). These data demonstrate significant cognitive and memory decline in mice undergoing repeated cerebral ischemia-reperfusion.

### Plasma TMAO levels are elevated in mice subjected to repeated cerebral ischemia-reperfusion

**Figure 2:**
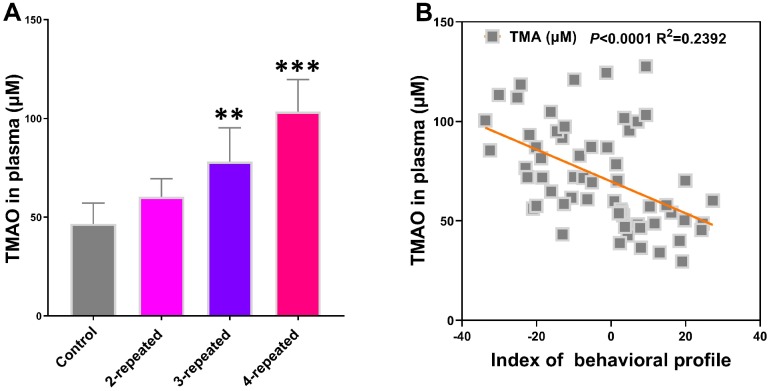
**Repeated cerebral ischemia-reperfusion in mice increases plasma TMAO levels.** (**A**) LC/MS analysis of plasma TMAO levels in the control and repeated cerebral ischemia-reperfusion model group mice are shown. (**B**) Spearman analysis shows the association between plasma TMAO levels and the behavioral profile of control and repeated cerebral ischemia-reperfusion model group mice. Note: ** denotes *P*<0.01 and *** denotes *P*<0.001 when compared with the control mice using one-way ANOVA analysis followed by Dunnett`s post hoc test. All the values are expressed as means ± S.D. Each group had 15 mice (n=15).

Liquid chromatography-tandem mass spectrometry (LC/MS) analysis showed that the plasma TMAO levels were significantly higher in the model group mice compared to the control group (Figure 2A). Furthermore, Spearman correlation analysis showed negative association between plasma TMAO levels and the behavioral as well as the electrophysiological characteristics (Figure 2B). These results demonstrate that elevated plasma TMAO levels correlate with behavioral and electrophysiological deficits in the model group mice.

### Baicalin supplementation suppresses cognitive, memory, and LTP decline induced by repeated cerebral ischemia-reperfusion

**Figure 3:**
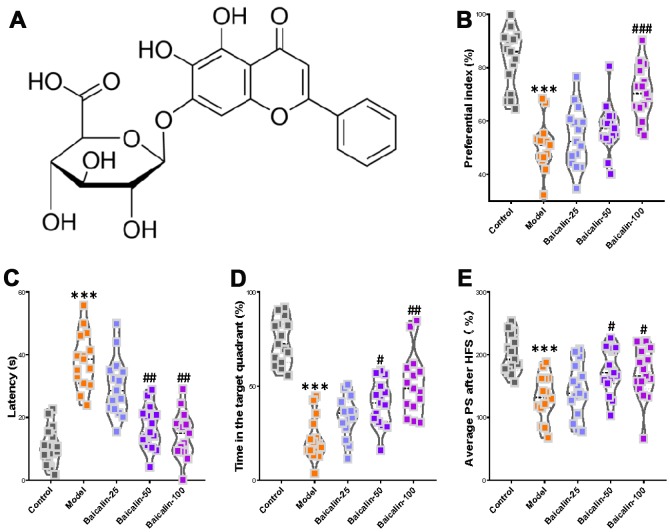
**Oral baicalin supplementation suppresses decline in cognition, memory, and LTP in repeated cerebral ischemia-reperfusion model mice.** (**A**) The chemical structure of baicalin. (**B**–**D**) The preferential index of the (**B**) novel object recognition test after 1h training, (**C**) latency in the testing phase, and (**D**) time spent in the target quadrant of Morris water maze test of control, repeated cerebral ischemia-reperfusion (model group), and 50 mg/kg and 100 mg/kg baicalin-treated model group mice is shown. (**E**) The average population spike amplitudes from all control, model mice, and 50 mg/kg and 100 mg/kg baicalin-treated model group mice is shown. Note: *** denotes *P*<0.001 compared with the control mice using unpaired Student`s *t*-tests; ^#^ denotes *P*<0.05, ^##^ denotes *P*<0.01, ^###^ denotes *P*<0.001 in comparison with the model mice using one-way ANOVA analysis followed by Dunnett`s post hoc test or a two-way repeated-measures ANOVA with post-hoc Tukey multiple comparisons test. All the values are expressed as means ± S.D. Each group had 15 mice (n=15).

We then examined whether baicalin supplementation (Figure 3A) improves cognition, memory, and LTP declines in mice that underwent carotid occlusion thrice to induce cerebral ischemia-reperfusion (model group). After a 7-day baicalin treatment following ischemia-reperfusion, object recognition test results showed significantly lower preferential index in the 100 mg/kg baicalin-treated model group mice after 1h training compared to the model group mice (Figure 3B). Morris water maze test results showed significantly longer escape latencies in the model group mice compared to the control group mice and the baicalin-treated model group mice (Figure 3C). The time in the target quadrant was significantly shorter for the model group mice compared to the control group, and the baicalin-treated model group mice (Figure 3D). The number of plate crossings and swimming speed (data not shown) were comparable for the mice belonging to the baicalin-treated model group, model group alone, and the control group. The LTP test results showed the average PS was significantly higher in the control, and baicalin-treated model group mice compared to the model group (Figure 3E). These findings show that oral treatment with baicalin ameliorates the decline in short-term object recognition memory, spatial learning and memory, and LTP, in response to repeated cerebral ischemia-reperfusion injury.

### Baicalin restores functional connectivity in repeated cerebral ischemia-reperfusion model mice

**Figure 4:**
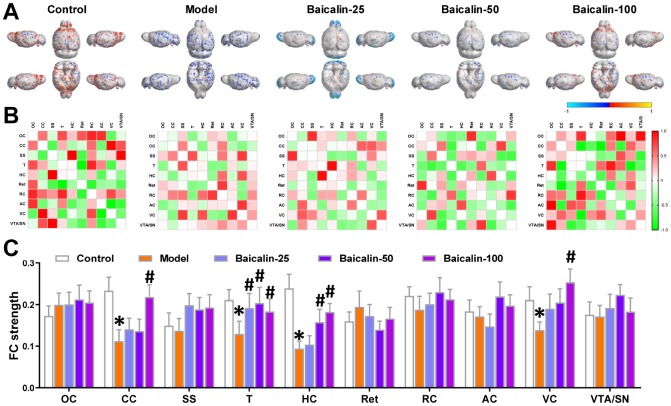
**Oral Baicalin supplementation restores brain functional connectivity in repeated cerebral ischemia-reperfusion model mice.** (**A**) Virtual graphics show brain functional connectivity (FC) in control, model, and baicalin-treated model group mice. (**B**) The mean FC matrices show the strength of functional connectivity between pairs of brain regions in control, model, and baicalin-treated model group mice. The color scale represents the strength of the functional connectivity. (**C**) The mean functional connectivity strength per brain network for control, model, and baicalin-treated model group mice is shown. Note: * denotes *P*<0.05 compared with the control mice using unpaired Student`s *t*-tests; ^#^ denotes *P*<0.05 compared with the model mice using two-way repeated-measures ANOVA with post-hoc Tukey multiple comparisons test. All the values are expressed as means ± S.D. Each group had 15 mice (n=15). The brain regions analyzed include orbitofrontal cortex (OC), cingulate cortex (CC), somatosensory cortex (SS), thalamus (T), hippocampus (H), retrosplenial cortex (Ret), rhinal cortex (RC), auditory cortex (AC), visual cortex (VC), and ventral tegmental area/substantia nigra (VTA/SN).

We then compared the brain regions of interest (ROI) based on the resting state-functional magnetic resonance imaging (rs-fMRI) results to evaluate the effects of baicalin treatment on the brain functional connectivity (FC). The ROIs were chosen based on the results of the rs-fMRI, and included orbitofrontal cortex (OC), cingulate cortex (CC), somatosensory cortex (SS), thalamus (T), hippocampus (HC), retrosplenial cortex (Ret), rhinal cortex (RC), auditory cortex (AC), the visual cortex (VC) and the ventral tegmental area/substantia nigra (VTA/SN). The functional connections between these ROIs are shown in the virtual graphs (Figure 4A) and correlation matrices (Figure 4B). The results show decreased FC in the cingulate cortex, thalamus, hippocampus, and visual cortex of model mice compared to the control mice (Figure 4C). The functional connectivity in the thalamus was significantly higher in the baicalin-treated model group mice for 3 different doses compared to the model group alone. FC in the hippocampus was significantly higher in the 50 and 100 mg/kg baicalin-treated model group compared to the model group. FC in the cingulate cortex and visual cortex was significantly higher in the 100 mg/kg baicalin-treated model group than the model group. These data indicate that baicalin improves FC in the brain regions of mice subjected to repeated cerebral ischemia-reperfusion. The effects were more pronounced for the model group mice treated with 100 mg/kg baicalin.

### Baicalin restores plasticity and dendritic spine density of hippocampal neurons in the repeated cerebral ischemia-reperfusion model mice

**Figure 5:**
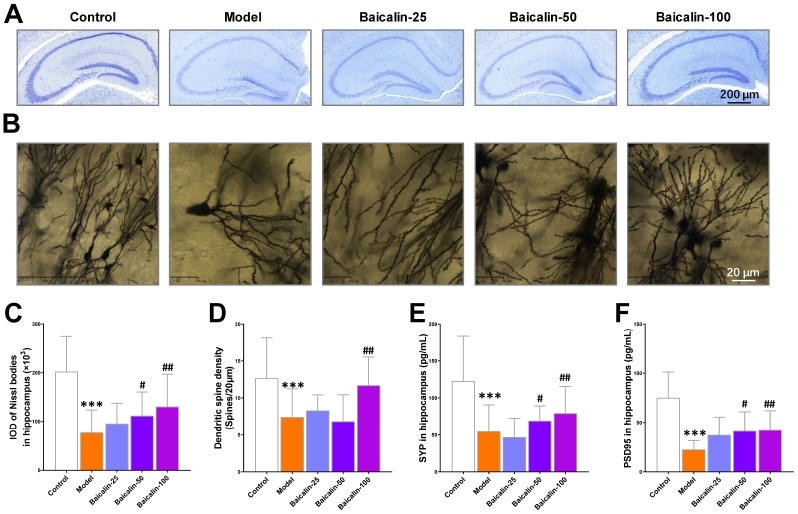
**Baicalin improves hippocampal neuronal plasticity in repeated cerebral ischemia-reperfusion model mice.** (**A**, **B**) Representative confocal microscopic images show (**A**) Nissl-stained and (**B**) golgi-stained hippocampal region of the brains of control, model, and Baicalin-treated model group mice. The brain tissue sections were subjected to Nissl and golgi staining, respectively. (**C**) The total number of Nissl bodies and (**D**) mean dendritic spine density of the hippocampal neurons based on the analysis of Nissl- and golgi-stained brain tissue sections of control, model, and Baicalin-treated model group mice are shown. (**E**, **F**) Representative images show immunohistochemical staining of (**E**) synaptophysin and (**F**) PSD95 proteins in the hippocampal region of control, model, and Baicalin-treated model group mice are shown. Note: *** denotes *P*<0.001 compared to the control mice using unpaired Student`s *t*-tests; ^#^ denotes *P*<0.05 and ^##^ denotes *P*<0.01 compared to the model mice using one-way ANOVA and the Dunnett`s post hoc test or a two-way repeated-measures ANOVA and the post-hoc Tukey multiple comparisons test. All the values are expressed as means ± S.D. Each group had 15 mice (n=15).

We performed Nissl and Golgi staining of brain sections to estimate Nissl bodies and dendritic spine density and determine the effects of baicalin on hippocampus neurons. Both Nissl and golgi staining showed neuropathological changes in the hippocampal region of the brain in the model group mice (Figure 5A and 5B). The model group mice showed significant reduction in the Nissl bodies in the hippocampal region compared to the control group (Figure 5C). Moreover, treatment with 50 and 100 mg/kg baicalin significantly increased the number of Nissl bodies in a dose-dependent manner in the model group mice (Figure 5C). Golgi staining analysis showed significant reduction in the dendritic spine density in the hippocampus of model group mice compared to the control group mice, but, treatment with 100 mg/kg baicalin significantly restored the dendritic spin density (Figure 5D). Immunohistochemical staining results showed that the levels of synaptic proteins, synaptophysin (SYP) and PSD95 were significantly reduced in the model group mice compared with the controls, but, their expression was significantly restored by treatment with 50 and 100 mg/kg baicalin (Figure 5E, 5F). These data demonstrate that baicalin significantly restores the hippocampal neuronal plasticity in mice subjected to repeated cerebral ischemia-reperfusion injury.

### Baicalin remodels the gut microbiota in mice subjected to repeated cerebral ischemia-reperfusion

**Figure 6:**
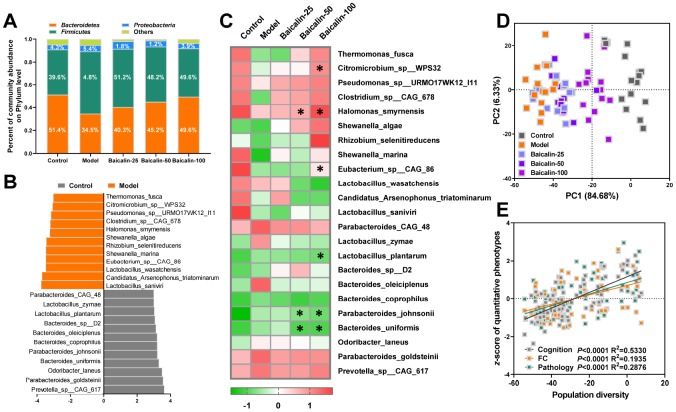
**Baicalin remodels gut microbiota in repeated cerebral ischemia-reperfusion model mice.** (**A**) Metagenomic assay analysis of the stool samples to determine the phyla of gut microbiota in control, model, and Baicalin-treated model group mice. (**B**) The most abundant gut microbial species levels in control, model, and baicalin-treated model group mice, as analyzed by linear discriminant analysis (LDA) coupled with effect size measurements are shown. The enriched taxa in the model and control group mice are indicated based on negative (orange) and positive (gray) scores, respectively. Also shown is the significant threshold LDA value of >3. (**C**) Heat map of the metagenomic analyses of gut microbial species in the stool samples of control and model group mice are shown. The red and green color codes represent high and low values, respectively. (**D**) Principal component analysis (PCA) scores of the gut microbial species in the stool samples of control and model group mice are shown. (**E**) Spearman correlation analysis shows the association between PCA scores and the behavioral profile, brain FC strength, and hippocampal neuron spasticity in control and model group mice. Note: * denotes *P*<0.05 compared with the model mice using one-way ANOVA analysis followed by Dunnett`s post hoc test. All the values are expressed as means ± S.D. Each group had 15 mice (n=15).

The gut microflora metabolizes dietary choline into TMA, which is the substrate for TMAO biosynthesis in the liver. We performed metagenomic analysis to determine the intestinal microbial populations at the phylum and species levels in different groups of mice. The gut microbial composition in the control group mice included 50.9% *Bacteroidetes*, 40.5% *Firmicutes*, and 4.6% *Proteobacteria.* The relative abundance of *Firmicutes* increased and the percentage of *Bacteroidetes* decreased in the model group mice, but, treatment with 50 and 100 mg/kg baicalin restored the levels of *Firmicutes* and *Bacteroidetes* to normal (Figure 6A). Species-level analysis revealed decreased abundance of *Thermomonas_ fusca, Citromicrobium_sp__WPS32, Pseudomonas_sp__ URMO17WK12_I11, Clostridium_sp__CAG_678, Halomonas_smyrnensis, Shewanella_algae, Rhizobium_ selenitireducens, Shewanella_marina, Eubacterium_sp__ CAG_86, Lactobacillus_wasatchensis, Candidatus_ Arsenophonus_triatominarum,* and *Lactobacillus_saniviri* in the model group mice compared to the controls (Figure 6B). Furthermore, repeated cerebral ischemia-reperfusion increased the relative abundance of *Parabacteroides_ CAG_48, Lactobacillus_zymae, Lactobacillus_plantarum, Bacteroides_sp__D2, Bacteroides_oleiciplenus, Bacteroides_coprophilus, Parabacteroides_johnsonii, Bacteroides_uniformis, Odoribacter_laneus, Parabacteroides_goldsteinii,* and *Prevotella_sp__CAG_ 617* compared with the controls (Figure 6B). The treatment of model group mice with 50 or 100 mg/kg baicalin significantly increased the relative abundance of *Halomonas_smyrnensis* and decreased the relative abundance of *Parabacteroides_johnsonii* and *Bacteroides_uniformis*; moreover, model group mice treated with 100 mg/kg baicalin showed increased abundance of *Citromicrobium_sp_WPS32* and *Eubacterium_sp_CAG_86* and decreased levels of *Lactobacillus_plantarum* (Figure 6B). We performed PCA to determine the influence of baicalin treatment on the gut microbial populations at the species level. The plot of PC1 vs. PC2 showed that each group formed a distinct cluster (Figure 6C). Spearman correlation analysis demonstrated that gut microbial remodeling correlated with cognition, memory and LTP decline, functional connectivity, and hippocampal neuronal plasticity in response to cerebral ischemia-reperfusion as well as baicalin treatment (Figure 6D). These results imply that baicalin remodels and restores the gut microbiota in the cerebral ischemia-reperfusion mice.

### Baicalin reduces TMA and TMAO synthesis in mice subjected to repeated cerebral ischemia-reperfusion

**Figure 7:**
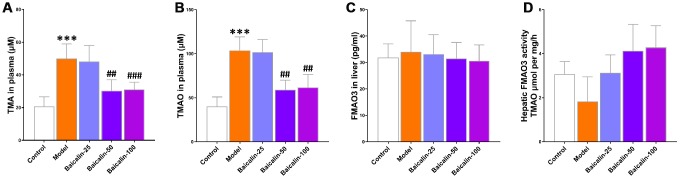
**Baicalin suppresses TMAO synthesis in repeated cerebral ischemia-reperfusion model mice.** The plasma levels of (**A**) TMA and (**B**) TMAO in control, model, and baicalin-treated model group mice are shown. (**C**) The hepatic FMO3 expression and (**D**) hepatic flavin-containing monoxygeneases (FMO) activity in the control, model, and baicalin-treated model group mice are also shown. Note: *** denotes *P*<0.001 compared with the control mice using unpaired Student`s *t*-tests; ^##^ denotes *P*<0.01 and ^###^ denotes *P*<0.001 compared with the model mice using one-way ANOVA analysis followed by Dunnett`s post hoc test. All the values are expressed as means ± S.D. Each group had 15 mice (n=15).

Next, we analyzed the effects of baicalin on the plasma levels and synthesis of TMA and TMAO. Our results show that 50 and 100 mg/kg baicalin administration significantly reduced the levels of TMA and TMAO, but, did not alter the levels of liver FMO3 protein and the activity of hepatic FMO (Figure 7). These results suggest that the gut microbes modulate the TMAO levels in response to repeated cerebral ischemia-reperfusion and baicalin treatments.

### Baicalin restores plasma clusterin levels and suppresses neuroinflammation in repeated cerebral ischemia-reperfusion model mice

**Figure 8:**
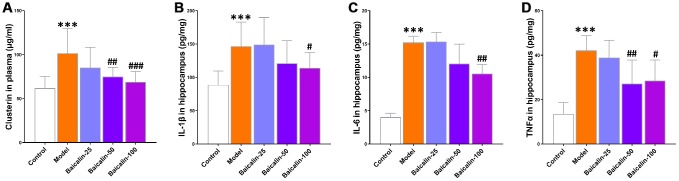
**Baicalin decreases plasma clusterin levels and pro-inflammatory cytokines in the hippocampus of repeated cerebral ischemia-reperfusion model mice.** (**A**) The plasma levels of clusterin, and (**B**) interleukin (IL)-1β, (**C**) IL-6, and (**D**) tumor necrosis factor α (TNFα) levels in the hippocampus of control, model, and baicalin-treated model group mice are shown. Note: *** denotes *P*<0.001 compared with the control mice using unpaired Student`s *t*-tests; ^#^ denotes *P*<0.05,^##^ denotes *P*<0.01, and ^###^ denotes *P*<0.001 compared with the model mice using one-way ANOVA followed by Dunnett`s post hoc test. All the values are expressed as means ± S.D. Each group had 15 mice (n=15).

Previous studies show that elevated plasma levels of clusterin correlate with neuroinflammation in response to brain injury [17, 18]. The plasma clusterin levels were significantly higher in the model group mice compared with the control group, but, the levels were normalized in model group mice treated with 50 or 100 mg/kg baicalin (Figure 8A). Furthermore, neuroinflammation is associated with the brain-related pathology underlying cerebral ischemia-reperfusion. Clusterin is a biomarker for the immune response that follows cerebral ischemia-reperfusion [19]. Therefore, we evaluated the neuroinflammation status in the hippocampus and observed significantly higher levels of pro-inflammatory cytokines, IL-1β, IL-6, and TNF-α in the model group mice compared to the control group mice, but, the levels of these cytokines were restored to normal by treatment with 50 or 100 mg/kg baicalin (Figure 8B, 8D). This suggests that baicalin suppresses neuroinflammation in mice subjected to repeated cerebral ischemia-reperfusion.

### Baicalin suppresses cognition, memory, and LTP decline by remodeling gut microbiota in mice subjected to repeated cerebral ischemia-reperfusion

**Figure 9:**
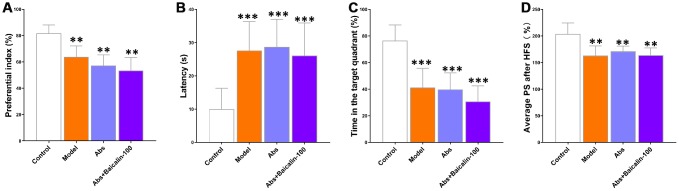
**Baicalin improves cognition and LTP in repeated cerebral ischemia-reperfusion model mice via remodeling of gut microbiota.** (**A**) Preferential index in the testing phase of the novel object recognition test after 1h training, (**B**) latency in the testing phase, (**C**) time spent in the target quadrant during the Morris water maze test, and (**D**) average population spike amplitudes are shown for control, model group mice treated with antibiotics, and model group mice treated with antibiotics plus baicalin. Note: ** denotes *P*<0.01 and *** denotes *P*<0.001 compared with the control mice using one-way ANOVA analysis followed by the Dunnett`s post hoc test. All the values are expressed as means ± S.D. Each group had 15 mice (n=15).

Next, we treated the model group mice with broad-spectrum antibiotics (Abs) with or without baicalin for 7 days to deplete the gut microbes. The model group mice treated with antibiotics showed significantly lower preferential index (Figure 9A), longer escape latencies (Figure 9B), shorter time in the target quadrant (Figure 9C) and decreased average PS (Figure 9D) compared to the untreated model group mice. We also observed that antibiotic treatment diminished the improvements in cognition and LTP shown by the bacialin-treated model group mice (Figure 9A–9D). These results demonstrate that baicalin induced remodeling of the gut microbiome is required to suppress pathological changes in brain functions, including cognition, memory, and LTP declines in mice subjected to repeated cerebral ischemia-reperfusion.

## DISCUSSION

In the current study, we investigated the neuroprotective effects of baicalin in mice subjected to repeated cerebral ischemia-reperfusion. Our study demonstrates that baicalin (50 and 100 mg/kg) decreases the plasma levels of TMAO by modifying the composition of the intestinal microbiota. Subsequently, low TMAO levels attenuate neuroinflammation and improve cognition, short-term memory, LTP, and plasticity of hippocampal neurons. Our study also demonstrates that the neuroprotective effects of baicalin involves remodeling of the gut microbiota.

Meta-organismal metabolism of dietary choline generates TMAO, which modulates cholesterol and sterol metabolism, and is associated with adverse cardiovascular outcomes [20]. Intestinal microbes metabolize l-carnitine into TMA, which is subsequently oxidized in the liver to TMAO by the members of the FMO family. Therefore, the gut flora regulates the production of TMAO, which is involved in the pathogenesis of several human diseases [5]. Depletion of gut microbiota with broad-spectrum antibiotics attenuates atherosclerosis by reducing the levels of TMA [21]. TMA agonist, DMB (3,3-dimethyl-1 butanol), inhibits TMA synthesis and suppresses TMAO-dependent formation of endogenous macrophage foam cells and atherosclerotic lesions [22]. Furthermore, suppression of FMO3 decreases circulating TMAO levels and reduces atherosclerosis [23]. TMAO is also an independent predictor of ischemic stroke [24]. In the current study, we demonstrate that increased plasma TMAO levels in the mice subjected to repeated cerebral ischemia-reperfusion correlate with significant decline in short-term object recognition memory, spatial learning ability and memory, and LTP.

Clusterin is an extracellular chaperone protein that inhibits stress-induced aggregation and precipitation of several proteins [25]. Clusterin levels are elevated in several neurodegenerative diseases such as amyotrophic lateral sclerosis, multiple sclerosis, transmissible spongiform encephalopathies, Alzheimer`s disease, and Huntington`s disease [26]. *Clusterin* knockout mice subjected to middle cerebral artery occlusion show a larger inflammatory reaction around the lesions than the wild-type mice, whereas the mice overexpressing clusterin display fewer inflammatory cells in the peri-infarct region and better recovery compared to the wild-type mice [27]. However, the role of clusterin in ischemia is controversial because several studies demonstrate that clusterin suppresses recovery from brain injury. For example, the mouse model of cerebral palsy shows significantly higher levels of clusterin in the dying neurons, whereas heterozygous and homozygous clusterin knockout mice show significantly lower level of brain lesions in an allele-dose-dependent manner [28]. Moreover, exogenous clusterin enhances cell death in mixed cultures of primary cortical neurons and astrocytes. These data indicate that clusterin modulates neuronal death following brain ischemia. However, it is not clear if these contradictory results of clusterin are because of differences in experimental conditions, including differences in the animal models and mouse strains, developmental stages, the extent of clusterin overexpression, the type of gene knockout, and the detection methods used to analyze ischemic brain damage. Furthermore, clusterin interacts with several components of the complement system and its expression is modulated by multiple pro-inflammatory cytokines [29]. Therefore, clusterin is a crucial indicator of the status of neuroinflammation induced by repeated cerebral ischemia-reperfusion. Our study demonstrates elevated levels of clusterin and pro-inflammatory cytokines in the cerebral ischemia-reperfusion mice, as shown in previous studies. Moreover, treatment with 100 mg/Kg bacailin significantly reduces the levels of pro-inflammatory cytokines, including IL-1β, IL-6, and TNF-α in the hippocampus of ischemia mice.

The limitation of this study is that we do not show a direct link between TMAO levels and clusterin function in the brain before and after bacailin administration. TMAO-specific receptor that modulates clusterin function has not yet been identified. Moreover, the effects of TMAO may be partly or wholly independent of clusterin. A report shows that TMAO acts as a small molecule protein chaperone that can efficiently bind and induce changes in protein conformation and stabilize protein folding directly [30]. Thus, it is conceivable that some of the effects of TMAO may be clusterin-independent, whereas other effects may occur through clusterin-dependent signaling pathways. These mechanisms need to be investigated further.

In conclusion, our results show that repeated cerebral ischemia-reperfusion changes composition of the gut microbiota and increases TMAO levels, which correlate with cognitive decline and other related neuropathology. Furthermore, oral baicalin supplementation protects from brain injury because of repeated cerebral ischemia-reperfusion by remodeling the gut microbiota, which decreases circulating TMAO and clusterin protein levels, thereby suppressing neuroinflammation and improving cognition, memory, LTP, and hippocampal neuronal plasticity. We postulate that oral baicalin therapy is a potential treatment for cerebral ischemia-reperfusion injuries.

## MATERIALS AND METHODS

### Animals

A randomized double-blind controlled study using 135 male C57BL/6J mice (2-3 months old) was conducted at the animal experimental center of Harbin medical university. Before bilateral common carotid artery occlusion, the animals were group-housed (four animals per cage) in enriched, individually ventilated cages. Standard food pellets and deionized water were available *ad libitum*. In the room where the mice were fed, there was a man-made 12-hour light-dark cycle, humidness control, and background noise. Room temperature was kept constant at 22 ± 2°C. After bilateral common carotid artery occlusion, the mice were kept separately to monitor the intake of the experimental diets by each individual mouse. Experiments were performed according to the references in the Guide for the Care and Use of Laboratory Animals published by the National Institutes of Health and were approved by the Institutional Animal Care and Use Committee of Harbin medical university. All behavioral and MRI experiments were performed in the animal experimental center of Harbin medical university between 8 a.m. and 6 p.m.

### Mouse model of repeated cerebral ischemia-reperfusion

We induced repeated global cerebral ischemia with the bilateral common carotid arteries method as previously described [31]. Briefly, mice were anesthetized with isoflurane (4%, inhalation) and xylazine (4 mg/kg, intraperitoneal injection). Then, maintained with isoflurane (1.5%) in 40% oxygen and 60% nitrous oxide and through a plastic mask. The body temperature was maintained at about 37.0 °C by a heating lamp during the course of the operation. A midline anterior incision in the neck was made to expose the bilateral common carotid arteries. Subsequently, each artery separated from the surrounding tissue and occluded with 3-0 silk for 20 min using microaneurysm clips and the cerebral blood flow (CBF) reduction was recorded by laser Doppler flowmetry (Omega Wave, Japan). The carotid occlusion was repeated two, three, or four times with a 10-min interval. Finally, anesthesia was closed, the skin incision was seamed, and the mice were replaced in the recovery cage. For sham surgery, the bilateral common carotid arteries were isolated using the same anesthetic and surgical procedure without obstructed blood flow.

### Group and treatment

Immediately after cerebral ischemia-reperfusion, mice were randomly divided into two experimental groups by a random sequence generator, with control group (sham surgery, n=15), 2-repeated group (occlusion was repeated 2 times, n=15), 3-repeated group (occlusion was repeated 3 times n=15), and 4-repeated group (occlusion was repeated 4 times, n=15). The behavioral experiment, electrophysiological experiment, and quantitation of flavin monooxygenase 3 (FMO3), Trimethylamine-N-oxide (TMAO) and trimethylamine (TMA) levels were performed at 7 days post-ischemia.

Baicalin (Sigma, USA; purity > 95%; MF, C21H18O11; MW, 446.36) (Figure 3A) was dissolved in physiological saline and administrated immediately after sham operation or ischemia (occlusion was repeated 3 times) on the first day. In the subsequent 7 days, all animals (except for sham surgery group and model group) were treated with the intragastric administration of baicalin (25 mg/kg, 50 mg/kg, or 100 mg/kg) between 8:30 and 11:00 each morning. Seventy-five C57BL/6J mice were randomly divided into five groups. In the sham surgery group (n=15) and model group (n=15), mice were administrated identically, with oral treatment, except that physiological saline (0.1 ml/100g) was instead of baicalin. In Baicalin-25 (n=15), Baicalin-50 (n=15), Baicalin-100 (n=15) groups, mice were respectively treated with 25 mg/kg, 50 mg/kg, or 100 mg/kg baicalin.

For Abs treatment, we fed male C57BL/6J mice (except for sham surgery group and model group) a supplemented with 100 mg/kg baicalin, in the absence or presence of 0.5 g/L vancomycin, 1 g/L neomycin sulfate, 1 g/L metronidazole, 1 g/L ampicillin (Sigma-Aldrich, USA) by daily oral gavage of 200 μL of the Abs solution for 7 day. Sixty C57BL/6J mice were randomly divided into four groups, including sham surgery group (n=15), model group (n=15), Abs group (n=15), Abs+ baicalin (n=15).

### Novel object recognition test

The testing pattern contains three phases: familiarization, training, and testing. The period of the experimental phases as detailed below. Briefly, to habituate them to the testing set, mice freely explored an empty chamber (5 min in daily) for 2 consecutive days. On the 3^rd^ day, animals explore two similar objects (sample objects A and B) that were uniformly positioned at opposing ends of the chamber. Object exploration was operationally defined as the time animals spent bodily contacting the object A/B within 0.2 cm. Each mouse was permitted to explore the objects for 5 min. After a 1-hour or 24-hour, one of the recognizable objects was replaced by a novel object (object C). The preferential index is analyzed to evaluate object recognition memory capacity in the 5-min testing period.

### Morris-water maze test

Morris-water maze test was progressed in a circular pool (90 cm in diameter and 45 cm deep). The pool was filled with water (about 35 cm of depth). The water temperature was kept at 20 ± 2°C by the addition of ice cube. The platform (6 cm in diameter) was situated 1-cm under the water surface. The training and testing periods were 60 s in duration. In the training period, animals accomplished four trials daily with a hidden platform for five consecutive days. Animals that could not get to the platform within 60 s were led up to the platform and positioned onto the platform for 10 s. In the testing period, the animal could search for the platform area for 60 s without the platform. The latency to find the hidden platform in experimental periods (training and testing periods) was analyzed.

### Electrophysiological experiment

The mice were anesthetized by 1.2 g/kg urethane i.p. and positioned in a stereotaxic fram. A bipolar stimulating electrode was interposed into the perforant path (anterior to lambda: 0.6 mm, midline: 2.4 mm, brain surface: 1.6-2.1 mm). The evoked potentials were obtained by a pole at the dentate gyrus (bregma: -2.0 mm, midline: 1.0 mm, brain surface: 1.7-2.2 mm). The electrostimulations generated by a stimulator (Nihon Kohden). The pulses (1/60 Hz, 0.1 ms) transmitted throughout an isolator (Nihon Kohden) to provide a stable current. The evoked responses were amplified and low-pass filtered (1000 Hz, Axon), then transmitted through an information acquisition system (DIGIDATA, Axon). After acquiring a steady stimulus-response curve, a 1/3-1/2 maximum population spike (PS) was applied. Following a 30-min recording, the long-term potentiation (LTP) was provoked by high-frequency stimulation (HFS), and the PSs were logged for 60 min (31-90 min).

### Magnetic resonance imaging procedures

MRI procedures were performed at 7 days post-ischemia on a 7.0 T Biospec small animal MRI system (Bruker BioSpin, Germany) with the Paravision 6.0 software platform (www.bruker.com). In short, the mice were anesthetized with 2% isoflurane, which was administered in a 2:1 oxygen and nitrous oxide mixture and placed in a stereotactic holder. The physiological state of all mice was supervised via the imaging procedure. An MR-compatible small animal monitoring and gating system were used to observe the breathing rate and an MR-compatible small animal heating system was used to maintain body temperature about 37.0°C. Images were obtained by a quadrature surface coil for mice and a standard Bruker cross coil with a quadrature volume coil. T2-weighted echo-planar imaging (EPI) sequence was used to acquire MRI data (repetition time=3150 ms, echo time=35 ms, echo train length=8, the field of view=100 mm × 100 mm, acquisition matrix=256 × 256, 28 slices of 0.4 mm, flip angle=90°).

### Resting-state functional MRI

To assess patterns of functional connectivity (FC), rs-fMRI was determined. Rs-fMRI signals were acquired by a T2-weighted single-shot EPI sequence (repetition time=2000 ms, echo time=15 ms, echo train length=48, the field of view=100 mm × 100 mm, acquisition matrix=64 × 64, slice thickness=0.4 mm, the spacing between slices=0.4 mm, slice number=8400, flip angle=90°). The rs-fMRI datasets were primarily realigned to the first image by a 6-parameter rigid-body transformation (along the x-, y-, and z-axes and on three rotation parameters pitch, roll, and yaw) and least-squares approach with statistical parametric mapping (SPM) mouse toolbox, which extended the functionality of SPM5 [32]. Subsequently, datasets of all subjects were normalized to a study-specific EPI template, which generated by linear affine and nonlinear diffeomorphic transformation based on mean EPI images of each mouse. Finally, in-plane spatial smoothing was done by a Gaussian kernel. The smoothed data and a mask for 10 region of interests (ROIs) were selected, including orbitofrontal cortex (OC), cingulate cortex (CC), somatosensory cortex (SS), thalamus (T), hippocampus (HC), retrosplenial cortex (Ret), rhinal cortex (RC), auditory cortex (AC), the visual cortex (VC) and the ventral tegmental are/substantia nigra (VTA/SN). All selected ROIs were loaded into the REST toolbox [33]. Meanwhile, to correct for a potential movement that generated during the scanning process, the motion parameters acquiring from the realignment were loaded as covariates. To reserve the low-frequency fluctuations of the time course of the blood-oxygenation-level-dependent (BOLD) signal, the filter was set between 0.01 and 0.08 Hz. The time courses were extracted for each ROI and correlation coefficients between the time courses of each ROI were computed. Then, these correlation coefficients were z-transformed by an in-house program developed in MATLAB 2013b and presented in a functional connectivity strength correlation matrix. These individual z-transformed zFC-maps were loaded in SPM12 and mean zFC-maps were computed per group. The mean zFC-maps are presented by overlaying them on an anatomical image.

### Nissl staining

The slices were stained by 0.7% cresyl violet acetate (Beyotime, China) and taken photos by a transmission electron microscope (Hitachi, Japan). The integrated optical density (IOD) of hippocampal Nissl bodies was analyzed by IPP 6.0 software.

### Golgi staining

The hemispheres were transported to the Golgi staining solution, for dendritic spine density investigation. Golgi staining was performed according to the instructions of the FD Rapid Golgistain™ Kits (FD Neuro Technologies, USA).

### Enzyme-linked immunosorbent assay

The concentrations of PSD95 and synaptophysin protein in the hippocampus were measured by DLG4 / PSD95 ELISA Kit (LS-F7142-1, LifeSpan BioSciences, USA) and ELISA Kit for synaptophysin (CEA425Mu-1, Lifeome BioLabs, USA), the level of FMO3 in the liver was measured by mouse flavin containing monooxygenase 3 ELISA Kit (MBS9327471, MyBioSource, USA), the level of clusterin in the plasma were measured by mouse clusterin quantikine ELISA kit (MCLU00, R&D Systems, USA), in accordance with the manufacturer`s instructions, respectively. The absorbance was detected at 450 nm via an Enspire^TM^ multilabel reader 2300 (PerkinElmer, Finland).

### Quantitation of TMAO and TMA concentrations

The 80 μl of 80% acetonitrile was added to 20 μl plasma for protein precipitation. The _d9_-(trimethyl) TMA and _d9_-(trimethyl) TMAO were added to plasma, as interior standards. Samples were centrifuged for 10 min (20,000 × g, 4°C) after 30 min, and then examined by stable isotope dilution liquid chromatography-tandem mass spectrometry (LC/MS) as previous description [34]. Concisely, LC/MS assay was carried out via an Agilent 6410 Triple Quad LC/MS (Agilent Technologies, USA). The capillary voltage was warmed to 350 °C and fit up at 4000 V. TMAO and TMA were assessed by electrospray ionization in positive-ion mode (*m/z* 77-58, and *m/z* 61-44) with multiple reaction monitoring. The plasma TMAO and TMA levels were measured by an Agilent HPLC system. Chromatographic separation was performed on an hydrop interaction liquid chromatography (HILIC) column (3.5μm internal diameter, 150 × 2.1 mm, WATERS) isolated by a flex capillary HILIC guard column (3.5μm internal diameter, 10 × 2.1 mm, WATERS). The mobile phase A (methanol with 0.1% formic acid), and phase B (water with 0.1% formic acid) were employed at a ratio of 30:70 with a flow velocity of 0.20 ml/min. The calibration curves were calculated by adding a variety of concentrations of TMA and TMAO standards to control plasma, which allowed us to calculate the concentrations of TMA and TMAO.

### Measurement of the enzymatic activity of FMO in the liver

The activity of the hepatic FMO enzyme was quantified on the basis of previously published methods with minor modifications [34]. In brief, 100 mM NADPH, 100 μM TMA, and 1 mg homogenate of hepatic protein were mixed in 250 μl reaction in 10 mM HEPES buffer (pH 7.4). The reaction was ended via 0.2 N formic acids after 8h, subsequently transiting by a 3 K spin filter, and instantly stored at -80 °C till the time of assessment. For analyzes, the inner standard with the thawed filter liquor was injected into LC/MS to calculate the oxidized product TMAO.

### Metagenomic analyses

All excrements were spontaneously defecated by animals and collected immediately. A total of 180-220 mg of fresh excrements were collected. Total genome DNA was obtained by the EZNA DNA Kit, and the concentration and purity of the obtained DNA were detected by a TBS-380 mini-fluorometer and NanoDrop 2000 spectrophotometer, respectively. The paired-end library was constructed by a TruSeqTM DNA Sample Prep Kit. Paired-end sequencing was accomplished on the Illumina HiSeq4000 platform (Illumina Inc., USA) and applying the HiSeq 3000/4000 PE Cluster and SBS kits, according to instructions. Metagenomics data were assembled by MEGAHIT [35]. Contigs (length over 300 bp) were selected for final assembly and intended for subsequent gene prediction and annotation. Open reading frames of each assembled contig were predicted by MetaGene [36]. All predicted genes (greater than 95% sequence identity) were clustered by CD-HIT [37] and mapped to representative sequences by short oligonucleotide analysis package [38]. Characteristic sequences of non-redundant gene catalogs were matched to the NCBI NR database by the BLAST with an e-value cutoff of 1e^-5^ for taxonomic annotations [39].

### Multiplex bead analysis

The supernatant of hippocampus tissue was centrifuged according to the manufacturer instructions, and then diluted 1:1 in assay buffer and examined by a Luminex 200 (Luminex, USA). The protein concentration of interleukin-1β (IL-1β), IL-2, IL-4, IL-5, IL-6, IL-17, interferon-induced protein 10 (IP-10), granulocyte colony-stimulating factor (G-CSF), tumor necrosis factor α (TNF-α), granulocyte-macrophage colony-stimulating factor (GM-CSF), interferon-γ (IFNγ), Eotaxin, regulated upon activation normal T cell expressed and secreted factor (RANTES), monocyte chemotactic protein-1 (MCP-1), macrophage inflammatory protein-1β (MIP-1β) were detected by a multiplex map kit (Mouse Cytokine/Chemokine Magnetic Bead Panel-Immunology Multiplex Assay MCYTOMAG-70K, Millipore).

### Statistical analyses

All data were means ± S.D. The part data was plotted and analyzed part by GraphPad Prism 8.0. Comparison of data between two groups was performed by Student`s *t*-tests. Data from the multiple groups were compared by a one-way analysis of variance (ANOVA) followed by a Dunnett`s post hoc test or a two-way repeated-measures ANOVA with post-hoc Tukey multiple comparisons test. The correlations between TMAO levels in plasma and cognitive performance or pathology index were measured by Spearman correlation coefficients (R, v3.1.2). Principal component analysis plot was also calculated and generated by R. Results were considered statistically significant when *P* <0.05.

### Ethical approval

Animal experiments were processed in accordance with the references in the Guide for the Care and Use of Laboratory Animals published by the National Institutes of Health and were approved by the Institutional Animal Care and Use Committee of Harbin Medical University.

## References

[1] Go AS, Mozaffarian D, Roger VL, Benjamin EJ, Berry JD, Borden WB, Bravata DM, Dai S, Ford ES, Fox CS, Franco S, Fullerton HJ, Gillespie C, et al, American Heart Association Statistics Committee and Stroke Statistics Subcommittee. Executive summary: heart disease and stroke statistics--2013 update: a report from the American Heart Association. Circulation. 2013; 127:143–52. 10.1161/CIR.0b013e318282ab8f 23283859

[2] Rothwell PM, Algra A, Amarenco P. Medical treatment in acute and long-term secondary prevention after transient ischaemic attack and ischaemic stroke. Lancet. 2011; 377:1681–92. 10.1016/S0140-6736(11)60516-3 21571151

[3] Xian Y, Federspiel JJ, Hernandez AF, Laskowitz DT, Schwamm LH, Bhatt DL, Smith EE, Fonarow GC, Peterson ED. Use of Intravenous Recombinant Tissue Plasminogen Activator in Patients With Acute Ischemic Stroke Who Take Non-Vitamin K Antagonist Oral Anticoagulants Before Stroke. Circulation. 2017; 135:1024–35. 10.1161/CIRCULATIONAHA.116.023940 28119380

[4] Sawaguchi Y, Wang Z. Ultrasound Acceleration of rt-PA Thrombolysis Depends on Acoustic Intensity. Biol Pharm Bull. 2017; 40:97–103. 10.1248/bpb.b16-00702 27773879

[5] Tilg H. A Gut Feeling about Thrombosis. N Engl J Med. 2016; 374:2494–96. 10.1056/NEJMcibr1604458 27332910

[6] Winek K, Meisel A, Dirnagl U. Gut microbiota impact on stroke outcome: fad or fact? J Cereb Blood Flow Metab. 2016; 36:891–98. 10.1177/0271678X16636890 26945017 PMC4853845

[7] Chen Y, Liang J, Ouyang F, Chen X, Lu T, Jiang Z, Li J, Li Y, Zeng J. Persistence of Gut Microbiota Dysbiosis and Chronic Systemic Inflammation After Cerebral Infarction in Cynomolgus Monkeys. Front Neurol. 2019; 10:661. 10.3389/fneur.2019.00661 31316450 PMC6611357

[8] Rahmati H, Momenabadi S, Vafaei AA, Bandegi AR, Mazaheri Z, Vakili A. Probiotic supplementation attenuates hippocampus injury and spatial learning and memory impairments in a cerebral hypoperfusion mouse model. Mol Biol Rep. 2019; 46:4985–95. 10.1007/s11033-019-04949-7 31286392

[9] Singh V, Roth S, Llovera G, Sadler R, Garzetti D, Stecher B, Dichgans M, Liesz A. Microbiota Dysbiosis Controls the Neuroinflammatory Response after Stroke. J Neurosci. 2016; 36:7428–40. 10.1523/JNEUROSCI.1114-16.2016 27413153 PMC6705544

[10] Benakis C, Brea D, Caballero S, Faraco G, Moore J, Murphy M, Sita G, Racchumi G, Ling L, Pamer EG, Iadecola C, Anrather J. Commensal microbiota affects ischemic stroke outcome by regulating intestinal γδ T cells. Nat Med. 2016; 22:516–23. 10.1038/nm.4068 27019327 PMC4860105

[11] Winek K, Dirnagl U, Meisel A. The Gut Microbiome as Therapeutic Target in Central Nervous System Diseases: implications for Stroke. Neurotherapeutics. 2016; 13:762–74. 10.1007/s13311-016-0475-x 27714645 PMC5081128

[12] Winek K, Engel O, Koduah P, Heimesaat MM, Fischer A, Bereswill S, Dames C, Kershaw O, Gruber AD, Curato C, Oyama N, Meisel C, Meisel A, Dirnagl U. Depletion of Cultivatable Gut Microbiota by Broad-Spectrum Antibiotic Pretreatment Worsens Outcome After Murine Stroke. Stroke. 2016; 47:1354–63. 10.1161/STROKEAHA.115.011800 27056982 PMC4839545

[13] Wang ZL, Wang S, Kuang Y, Hu ZM, Qiao X, Ye M. A comprehensive review on phytochemistry, pharmacology, and flavonoid biosynthesis of Scutellaria baicalensis. Pharm Biol. 2018; 56:465–84. 10.1080/13880209.2018.1492620 31070530 PMC6292351

[14] Wang P, Cao Y, Yu J, Liu R, Bai B, Qi H, Zhang Q, Guo W, Zhu H, Qu L. Baicalin alleviates ischemia-induced memory impairment by inhibiting the phosphorylation of CaMKII in hippocampus. Brain Res. 2016; 1642:95–103. 10.1016/j.brainres.2016.03.019 27016057

[15] Li S, Sun X, Xu L, Sun R, Ma Z, Deng X, Liu B, Fu Q, Qu R, Ma S. Baicalin attenuates in vivo and in vitro hyperglycemia-exacerbated ischemia/reperfusion injury by regulating mitochondrial function in a manner dependent on AMPK. Eur J Pharmacol. 2017; 815:118–126. 10.1016/j.ejphar.2017.07.041 28743390

[16] Ju M, Liu Y, Li M, Cheng M, Zhang Y, Deng G, Kang X, Liu H. Baicalin improves intestinal microecology and abnormal metabolism induced by high-fat diet. Eur J Pharmacol. 2019; 857:172457. 10.1016/j.ejphar.2019.172457 31202804

[17] Iłżecka J, Iłżecki M, Grabarska A, Dave S, Feldo M, Zubilewicz T. Clusterin as a potential marker of brain ischemia-reperfusion injury in patients undergoing carotid endarterectomy. Ups J Med Sci. 2019; 124:193–98. 10.1080/03009734.2019.1646359 31460820 PMC6758642

[18] Troakes C, Smyth R, Noor F, Maekawa S, Killick R, King A, Al-Sarraj S. Clusterin expression is upregulated following acute head injury and localizes to astrocytes in old head injury. Neuropathology. 2017; 37:12–24. 10.1111/neup.12320 27365216

[19] Dabrowska S, Andrzejewska A, Lukomska B, Janowski M. Neuroinflammation as a target for treatment of stroke using mesenchymal stem cells and extracellular vesicles. J Neuroinflammation. 2019; 16:178. 10.1186/s12974-019-1571-8 31514749 PMC6743114

[20] Zhu W, Gregory JC, Org E, Buffa JA, Gupta N, Wang Z, Li L, Fu X, Wu Y, Mehrabian M, Sartor RB, McIntyre TM, Silverstein RL, et al. Gut Microbial Metabolite TMAO Enhances Platelet Hyperreactivity and Thrombosis Risk. Cell. 2016; 165:111–24. 10.1016/j.cell.2016.02.011 26972052 PMC4862743

[21] Yin J, Liao SX, He Y, Wang S, Xia GH, Liu FT, Zhu JJ, You C, Chen Q, Zhou L, Pan SY, Zhou HW. Dysbiosis of Gut Microbiota With Reduced Trimethylamine-N-Oxide Level in Patients With Large-Artery Atherosclerotic Stroke or Transient Ischemic Attack. J Am Heart Assoc. 2015; 4. 10.1161/JAHA.115.002699 26597155 PMC4845212

[22] Wang Z, Roberts AB, Buffa JA, Levison BS, Zhu W, Org E, Gu X, Huang Y, Zamanian-Daryoush M, Culley MK, DiDonato AJ, Fu X, Hazen JE, et al. Non-lethal Inhibition of Gut Microbial Trimethylamine Production for the Treatment of Atherosclerosis. Cell. 2015; 163:1585–95. 10.1016/j.cell.2015.11.055 26687352 PMC4871610

[23] Shih DM, Wang Z, Lee R, Meng Y, Che N, Charugundla S, Qi H, Wu J, Pan C, Brown JM, Vallim T, Bennett BJ, Graham M, et al. Flavin containing monooxygenase 3 exerts broad effects on glucose and lipid metabolism and atherosclerosis. J Lipid Res. 2015; 56:22–37. 10.1194/jlr.M051680 25378658 PMC4274068

[24] Liang Z, Dong Z, Guo M, Shen Z, Yin D, Hu S, Hai X. Trimethylamine N-oxide as a risk marker for ischemic stroke in patients with atrial fibrillation. J Biochem Mol Toxicol. 2019; 33:e22246. 10.1002/jbt.22246 30370581

[25] Garcia-Huerta P, Bargsted L, Rivas A, Matus S, Vidal RL. ER chaperones in neurodegenerative disease: folding and beyond. Brain Res. 2016; 1648:580–87. 10.1016/j.brainres.2016.04.070 27134034

[26] Yeh FL, Hansen DV, Sheng M. TREM2, Microglia, and Neurodegenerative Diseases. Trends Mol Med. 2017; 23:512–33. 10.1016/j.molmed.2017.03.008 28442216

[27] Wehrli P, Charnay Y, Vallet P, Zhu G, Harmony J, Aronow B, Tschopp J, Bouras C, Viard-Leveugle I, French LE, Giannakopoulos P. Inhibition of post-ischemic brain injury by clusterin overexpression. Nat Med. 2001; 7:977–79. 10.1038/nm0901-977 11533682

[28] Huang Z, Cheng C, Jiang L, Yu Z, Cao F, Zhong J, Guo Z, Sun X. Intraventricular apolipoprotein ApoJ infusion acts protectively in Traumatic Brain Injury. J Neurochem. 2016; 136:1017–25. 10.1111/jnc.13491 26670094

[29] Akison LK, Kuo J, Reid N, Boyd RN, Moritz KM. Effect of Choline Supplementation on Neurological, Cognitive, and Behavioral Outcomes in Offspring Arising from Alcohol Exposure During Development: A Quantitative Systematic Review of Clinical and Preclinical Studies. Alcohol Clin Exp Res. 2018; 42:1591–611. 10.1111/acer.13817 29928762

[30] Wilson MR, Zoubeidi A. Clusterin as a therapeutic target. Expert Opin Ther Targets. 2017; 21:201–13. 10.1080/14728222.2017.1267142 27978767

[31] Salehpour F, Farajdokht F, Mahmoudi J, Erfani M, Farhoudi M, Karimi P, Rasta SH, Sadigh-Eteghad S, Hamblin MR, Gjedde A. Photobiomodulation and Coenzyme Q(10) Treatments Attenuate Cognitive Impairment Associated With Model of Transient Global Brain Ischemia in Artificially Aged Mice. Front Cell Neurosci. 2019; 13:74. 10.3389/fncel.2019.00074 30983970 PMC6434313

[32] Sawiak SJ, Wood NI, Williams GB, Morton AJ, Carpenter TA. Voxel-based morphometry in the R6/2 transgenic mouse reveals differences between genotypes not seen with manual 2D morphometry. Neurobiol Dis. 2009; 33:20–27. 10.1016/j.nbd.2008.09.016 18930824

[33] Song XW, Dong ZY, Long XY, Li SF, Zuo XN, Zhu CZ, He Y, Yan CG, Zang YF. REST: a toolkit for resting-state functional magnetic resonance imaging data processing. PLoS One. 2011; 6:e25031. 10.1371/journal.pone.0025031 21949842 PMC3176805

[34] Bennett BJ, de Aguiar Vallim TQ, Wang Z, Shih DM, Meng Y, Gregory J, Allayee H, Lee R, Graham M, Crooke R, Edwards PA, Hazen SL, Lusis AJ. Trimethylamine-N-oxide, a metabolite associated with atherosclerosis, exhibits complex genetic and dietary regulation. Cell Metab. 2013; 17:49–60. 10.1016/j.cmet.2012.12.011 23312283 PMC3771112

[35] Li D, Liu CM, Luo R, Sadakane K, Lam TW. MEGAHIT: an ultra-fast single-node solution for large and complex metagenomics assembly via succinct de Bruijn graph. Bioinformatics. 2015; 31:1674–76. 10.1093/bioinformatics/btv033 25609793

[36] Noguchi H, Park J, Takagi T. MetaGene: prokaryotic gene finding from environmental genome shotgun sequences. Nucleic Acids Res. 2006; 34:5623–30. 10.1093/nar/gkl723 17028096 PMC1636498

[37] Fu L, Niu B, Zhu Z, Wu S, Li W. CD-HIT: accelerated for clustering the next-generation sequencing data. Bioinformatics. 2012; 28:3150–52. 10.1093/bioinformatics/bts565 23060610 PMC3516142

[38] Li R, Li Y, Kristiansen K, Wang J. SOAP: short oligonucleotide alignment program. Bioinformatics. 2008; 24:713–14. 10.1093/bioinformatics/btn025 18227114

[39] Altschul SF, Madden TL, Schäffer AA, Zhang J, Zhang Z, Miller W, Lipman DJ. Gapped BLAST and PSI-BLAST: a new generation of protein database search programs. Nucleic Acids Res. 1997; 25:3389–402. 10.1093/nar/25.17.3389 9254694 PMC146917

